# Malaria vaccines: facing unknowns

**DOI:** 10.12688/f1000research.22143.1

**Published:** 2020-04-27

**Authors:** Nirianne Marie Q. Palacpac, Toshihiro Horii

**Affiliations:** 1 Department of Malaria Vaccine Development, Research Institute for Microbial Diseases, Osaka University, Osaka, Japan

**Keywords:** malaria, vaccine, Plasmodium

## Abstract

Much of the gain in malaria control, in terms of regional achievements in restricting geographical spread and reducing malaria cases and deaths, can be attributed to large-scale deployment of antimalarial drugs, insecticide-treated bed nets, and early diagnostics. However, despite impressive progress, control efforts have stalled because of logistics, unsustainable delivery, or short-term effectiveness of existing interventions or a combination of these reasons. A highly efficacious malaria vaccine as an additional tool would go a long way, but success in the development of this important intervention remains elusive. Moreover, most of the vaccine candidate antigens that were investigated in early-stage clinical trials, selected partly because of their immunogenicity and abundance during natural malaria infection, were polymorphic or structurally complex or both. Likewise, we have a limited understanding of immune mechanisms that confer protection. We reflect on some considerable technological and scientific progress that has been achieved and the lessons learned.

## Introduction

*Plasmodium* protozoan parasites *P. falciparum*, *P. vivax*, *P. ovale* ( *P. ovale curtisi* and *P. ovale wallikeri*), *P. malariae*, and *P. knowlesi* can infect humans. *P. falciparum* is responsible for the majority of malaria-attributable deaths, and the global malaria burden is borne disproportionally by children in sub-Saharan Africa ^1^. Malaria symptoms range from severe headaches, chills, joint pain, and fever-like symptoms to red blood cell dysregulation and lysis that can lead to severe anemia, sequestration of infected red blood cells, or occlusion. Occlusion can lead to cerebral malaria or complications associated with placental malaria, including adverse birth outcomes with long-term sequelae or death.

Vaccines for malaria aim to either eliminate/prevent infection (for example, in the liver or blood or they interrupt transmission in mosquitoes) or control/limit parasite growth/multiplication and duration of infection ^2^. Approaches to malaria vaccine development have ranged from traditional (live-attenuated or killed) to subunit (single-antigen recombinant proteins that target the various stages in the parasite life cycle) vaccines to newer approaches targeting the inclusion of only defined antigenic regions or critical epitopes ^2– 4^. The struggle continues to develop a vaccine to protect people from this disease.

## Burden and current interventions

More than 90% of the sub-Saharan Africa population live in malaria-endemic areas ^1, 5^. In Africa, 99.7% of estimated malaria cases are due to *P. falciparum* ^1^. Outside of Africa, *P. vivax* accounts for most of the disease burden: 73% in the World Health Organization (WHO) Region of the Americas and 53% in the WHO Southeast Asia Region ^1^. *P. vivax* burden is difficult to assess as infections can cause relapse months or years after initial infection because of the persistence of hypnozoites in hepatocytes. Other species represent a small proportion globally. *P. ovale* and *P. malariae* are common sympatric species in malaria-endemic regions ^6, 7^. Data on the global burden of *P. knowlesi*, a zoonotic parasite, are much less known. Nevertheless, this species has been reported to cause severe human malaria and is important for certain populations in Indonesia, the Philippines, Cambodia, South Thailand, South Myanmar, and South Vietnam ^8– 10^.

The WHO World Malaria Report ^1^ and high-resolution maps for *P. falciparum* ^5^ and *P. vivax* ^11^ capture the considerable progress in reducing malaria burden and the stall in the rates of decline in recent years. In a handful of developed countries, control efforts were aided greatly by successful economic development, improved health-care systems, and urbanization ^5^. But in resource-poor settings, gains achieved are noted as fragile, relying on a limited number of interventions: insecticide-treated bed nets, indoor residual spraying, artemisinin combination therapy, intermittent preventive treatment, and increased access to diagnostic testing ^12^. Moreover, bottlenecks and gaps for optimal coverage exist because of less sensitive tools and infection/disease management; for example, pregnant women are possible reservoirs that sustain malaria transmission, and pyrimethamine is contraindicated in the first trimester ^13, 14^. The campaign for increased annual funding, from US $2.7 billion to US $6.4 billion, for investments that aimed to meet a 40% reduction in malaria incidence and mortality rates remains challenging ^15^. For vector control, four commonly used insecticide classes are already in widespread use in Africa, the Americas, Southeast Asia, the Eastern Mediterranean, and the Western Pacific, raising warning bells for a possible emergence of insecticide resistance ^12^. The same is true for antimalarial drugs. At present, there are only three major/broad categories of available antimalarials. Poor quality, incomplete treatment, and misuse (including continued use of artemisinin-based monotherapy) could result to development and spread of drug-resistant parasites. Fortunately, to date, no evidence for substantial drug resistance has been documented ^16– 18^. However, the Global Technical Strategy for Malaria 2016–2030 milestones ^15^, to reduce malaria incidence and mortality by at least 40% in 2020, cannot be attained with current tools.

## Malaria vaccines

By experience, vaccines are among the most successful and cost-effective public health tools. Millions of lives have been saved and a substantial reduction in morbidity has been associated with vaccine scale-up implementation against other diseases. A case in point would be the recent vaccine implementation of meningococcal vaccine in sub-Saharan Africa ^19^. A malaria vaccine, if implemented through the routine/existing immunization delivery programmes, such as the Expanded Programme on Immunization (EPI), could have more reaching coverage logistically and improved compliance (compared with traditional antimalarial prophylaxis) and would help close the gap left by other control measures. However, considerations for inclusion to the EPI also need validation in field trials. Co-administration of a candidate tuberculosis vaccine, MVA85A, with EPI vaccines resulted in lowered tuberculosis-specific immunogenicity ^20^. On the other hand, with the pre-erythrocytic malaria vaccine—chimpanzee adenovirus 63 and modified vaccinia virus Ankara encoding multiple epitope string thrombospondin-related adhesion protein (ChAd63 MVA ME-TRAP)—antibody responses to EPI vaccines were not altered in a phase Ib trial ^21^. Both studies underscore the importance of optimal schedules and timing of immunizations for testing in clinical trials as well as for actual vaccine implementation.

A number of vaccines in the malaria pipeline have similar challenges/limitations due in part to the complex biology and life cycle of *Plasmodium* and the immunological interplay between the parasite and host. At the same time, every malaria infection can be considered unique in terms of antigenic repertoire and host, frequency of exposure, age, access to treatment, and presence of co-morbidities. Three recent reviews summarize vaccines now in development ^2– 4^. RTS,S (registered as Mosquirix), based on the circumsporozoite protein, is thus far the only vaccine that has progressed beyond phase 3 and is now in a WHO-recommended and -sponsored pilot implementation program complementing a GlaxoSmithKline-sponsored phase 4 trial ^2^. RTS,S is a pre-erythrocytic vaccine directed at the sporozoite stage or at the infected hepatocyte. The vaccine, in theory, should prevent blood-stage infection but, in reality, proved to be “leaky” ^22^. The pilot implementation of RTS,S aims (a) to address whether the protection demonstrated in the 5- to 17-month old multicenter phase 3 trial can be replicated in the context of routine health systems with a four-dose schedule and to evaluate (b) the excess risk of febrile seizures observed within 7 days after a vaccine dose, (c) the number of meningitis cases, (d) the number of cerebral malaria cases in the malaria vaccine group compared with the control, and (e) the imbalance of mortality among girls who received the vaccine ^23^. The Joint Technical Expert Group (JTEG) on malaria vaccines also recommended monitoring the emergence of vaccine resistance strains and testing of alternative schedules and other strategies to improve the efficacy of RTS,S. The JTEG noted that, if safety concerns are resolved and favorable implementation data become available, the WHO can recommend country-wide introduction. Projection for RTS,S shows that if it is implemented in all 43 malaria-endemic countries in sub-Saharan Africa, 123 million (95% prediction interval, 117 to 129 million) malaria episodes over the first 10 years could be averted ^24^.

Indeed, the RTS,S experience is a learning curve for all other malaria vaccines in the pipeline. Of note, the concern about a possible negative effect on overall female mortality may indeed be valid and needs careful consideration since this finding was observed in both age groups ^25^ and in a number of vaccines ^26, 27^. Similar obstacles and lessons provide much hope, rather than pessimism, as we move forward in addressing some key challenges.

## Polymorphism

As in other vaccine candidates, polymorphism abrogates antigen recognition. On the other hand, conserved important epitopes are generally poorly immunogenic. In RTS,S, the dominant epitope recognized by antibodies—(NANP)n from the circumsporozoite protein (CSP)—is totally conserved in *P. falciparum* but the T-cell (as well as B-cell) epitopes are known to be polymorphic ^2, 3^. A number of other vaccine candidates did not show much promise after efficacy trials suggested strain-specific protection ^2– 4^. In fact, the lack of success in developing subunit blood-stage vaccine candidates and lack of cross-stage immunity were among the driving factors to develop whole parasite vaccines (WPVs). WPV supposes that no individual parasite protein can be sufficient to induce strain-transcending immunity and that, aside from antibodies, B and T cells also play a role. WPVs aimed to maximize antigen breadth, although, whether irradiated parasites, genetically attenuated parasites, chemically attenuated parasites or killed parasites, the vaccine pathway represents a significant challenge because of difficulties in vaccine manufacture and specific regulatory issues ^28^. So far, clinical trials have shown that WPVs are well tolerated in terms of reactogenicity ^2^. The presence of extraneous (possible toxins, pathogens, and contaminants) materials not essential for the generation of protective immune response as well as concerns on vaccine route, dose, and regimen are being addressed ^2, 3, 28^.

Other innovations in antigen discovery have now come up with an extensive list of potential antigens from genomic-, proteomic-, and transcriptomic-based approaches ^29– 32^. Similar to vaccine development against viral infections such as human immunodeficiency virus and influenza, structure-guided vaccine design is currently tapped to identify protective/functional and non-functional epitopes in antigen–antibody complexes ^33– 36^. With difficulties for representative *in vitro* models, structure-guided vaccine design has also been used to inform the development of at least the most promising vaccine candidate for *P. vivax*, the *Pv* Duffy-binding protein ^37, 38^.

These innovations have, likewise, pushed antigen delivery platforms. Since subunit vaccines based on soluble recombinant proteins are often poorly immunogenic, the use of virus capsid-like particle vaccine platforms is beginning in the malaria field ^39, 40^. Virus-like particles (VLPs), when overexpressed, spontaneously self-assemble into particles that are highly immunogenic because of their structural resemblance to original live viruses ^39– 43^. A direct comparison of three VLP platforms for a transmission-blocking vaccine ^44^ showed that high quantity and quality of antibodies with minimal reactogenicity could be achieved.

## Poor understanding of vaccine-induced immune response

To date, we still have a considerable lack of understanding in the immunological mechanisms that provide protection against malaria, including what immune responses are induced in people who are protected and in people who are not. Immune correlates of protection are known to exist for successful vaccines. This knowledge can permit licensure of new vaccine formulations or extension of vaccine indications to new populations on the basis of immunogenicity endpoints ^45, 46^. In some cases, evaluations are restricted to antibody titers and a few basic T-cell analyses: cytokine-ELISpot or interferon-gamma, interleukin-2 (IL-2), or tumor necrosis factor-alpha (TNFα) readouts or a combination of these. Hepatitis B vaccine testing was simplified when it was shown that hepatitis B S antigen antibodies of more than 10 mIU/mL were surrogate markers of protection ^47, 48^. Interestingly, a recent study also showed the utility of T-cell (TNFα, IL-10, or IL-6) responses 32 years after vaccination despite the absence of detectable anti-hepatitis B antibodies ^49^. The cellular responses provided insight on how to evaluate long-term immunity; even under natural exposure, no breakthrough hepatitis B virus (HBV) infection was recorded ^49^, suggesting that the immune response can be boosted to prevent acute illness and chronic HBV infection.

In malaria, antibody titers and T-cell responses showed limited correlation and have largely failed to predict vaccine efficacy (VE) or down-select vaccine formulations prior to late-stage, large trials ^50^. In RTS,S, antibody titer concentrations of 121 enzyme-linked immunosorbent assay units per milliliter (95% confidence interval [CI] 98–153) prevented 50% of infection, but no threshold level for protection was found in the phase 3 trial ^51^. Further review of previous trials also showed that a modification of dose and schedule improved vaccine protection: 62% of volunteers (10/16) given the full dose at the standard 0-, 1-, 2-month regimen were protected 3 weeks after the last vaccination, whereas 86% of volunteers (26/30) were protected when the third dose occurred 6 months after the second and the dose was reduced to one fifth of the original dose (known as fractional dosing ^52^). With a fractional dose, titers of anti-NANP antibody were similar to subjects vaccinated using standard regimen, and it was speculated that the altered dosing had an effect on antibody avidity, somatic hypermutation, and isotype switching ^52, 53^. The roles of CD4 ^+^ T-cell responses have been suggested but remained inconsistent ^54, 55^, although it cannot be denied that protection is indeed mediated by complex immune functions. The inability to maintain high antibody titer after vaccination also needs in-depth studies ^51^. Waning or duration of immunity remains an important question as current vaccine candidates still have to demonstrate an acceptable duration of protection so that frequent revaccination is not necessary.

Besides antibody responses, CD4 and T cells have also been implicated in the WPV, PfSPZ ^56^. The absence of a T-cell response in 6- to 11-month-old vaccinees was interpreted to mean that there will be no protection for this age group by using the current PfSPZ immunization regimen (three doses at 8-week intervals). For blood-stage vaccine GMZ2/alum, a phase 2 efficacy trial showed higher VE in children 3 to 4 years old (VE = 20%, 95% CI 4–33%) than in children 1 to 2 years old (VE = 14%, 95% CI 3.6–23%) ^57^. It was concluded that, compared with toddlers, the older children have antibodies that were more functionally competent in terms of avidity and IgG subclass profile. Acquired background immunity to malaria would have helped the older cohort.

There is general agreement that effective assessment of induced immune response needs unbiased, comprehensive profiling together with mathematical modelling/computational analyses. Such methods can capture the overall picture/combination effects of multiple immune responses ^45, 58, 59^. Robust screening assays to assess protective potential or detect non-direct inhibitory or neutralizing activity of antibodies or assays that capture antibody-complement interactions and their consequences to parasite function and viability are most important considerations. For meningococcal vaccines, the availability of the serum bactericidal antibody assay, which measures complement-mediated killing via functional antibody concentrations, has proven to be a valuable tool ^60^.

## Controlled human malaria infection

The licensing by US Food and Drug Administration of a live, oral cholera vaccine (Vaxchora) for use in travelers on the basis of controlled human infection model in volunteers 18 to 64 years of age raised hopes that a malaria vaccine for travelers could also utilize human challenge data and pass along the same regulatory approach for licensure ^61, 62^. Clearly, controlled human malaria infection (CHMI) is a powerful tool (a) to provide early-stage proof-of-concept efficacy for vaccine candidates and (b) to address the limitation of preclinical testing in mice and non-human primates to predict immune response and to study mechanisms of protective immunity ^63, 64^. Because this experimental model allows some homogeneity/control of parasitemia within and between treatment arms, a major advantage is the relatively small sample size required for the evaluation of VE. The model is also ideally useful to advance field trials of transmission-blocking vaccines ^65^. In addition, it is less restrictive to have access to blood samples for antibody and T-cell work, allowing in-depth studies.

So far, only RTS,S ^55^, chemoattenuated *P. falciparum* sporozoite vaccine ^66^, whole parasite immunization under chloroquine drug cover ^67^, and whole sporozoite radiation-attenuated vaccines ^68^ were able to induce sterile immunity in a significant proportion of human volunteers. CHMI was used by RTS,S to test for various formulations, adjuvants, and vaccine timings ^52, 55, 64^. The *P. vivax* CHMI model has been used successfully to test the efficacy of radiation-attenuated sporozoites administered by mosquito bite ^69^.

CHMI has been harnessed to identify the range and specificities of antibody responses and antigen targets ^70^ as well as down-select other vaccine candidates ^71^. Currently, five challenge strains are available for CHMI use ^72– 75^. This is important, as it is necessary to address the regulatory concern of strain-transcending protective efficacy or cross-strain protection. All strains so far show global geographic differences in genetic variation ^72, 73, 75^; however, how representative they are of the overall diversity of *P. falciparum* field population remains to be seen. Efforts are ongoing to increase familiarity on the restrictions and challenges of CHMI models to regulators to determine the feasibility to accept these trials as part of the regulatory package for licensure ^63, 64, 76, 77^.

## Low-level parasite infection and immune tolerance

Low-level parasite infection confuse diagnostics/detection or attribution of symptom/fever to malaria and raise the question of how the chronic nature of malaria infection (persistent parasite infection) amplifies or down-regulates immune response. B-cell hypo-responsiveness as a result of meningococcal and polysaccharide vaccines has been reported ^78^. Many clinical trials suggest that pre-existing immunity induced immune tolerance, resulting in lower immunogenicity and efficacy in African adults and malaria-exposed children ^79– 82^. The picture becomes more complicated when host genetics, access to treatment, and co-infection are considered. Performing studies to assess these confounding/contributory variables in vaccine trials are needed.

## Vaccine efficacy in clinical trials

Endpoints and assessment methods to measure VE in clinical trials also lack clear consensus. In malaria-endemic areas, the rate of acquisition of natural immunity will vary between individuals; even within a country, malaria parasite transmission differs per site depending on climate/season and breeding sites. These factors affect the clinical trial design, surveillance methods, follow-up, and size of cohort. With RTS,S, during clinical trials, VE was evaluated by measuring time to first infection since natural infection is present in malaria-endemic areas and there is no control of time or amount of exposure to malaria in the different subjects. However, the WHO/JTEG specifically requested that all vaccine efficacies be reported against all episodes of the outcome (not only first or only episode) to better reflect the public health contribution of the vaccine. And what about boosting? Boosting through natural infection appears to be an important contributor in reducing the multiplicity of malaria episodes, especially for the blood-stage vaccine, SE36 ^83^. Of note, not all infections are clinically important (or manifest as clinical symptoms) and this complicates predictions for exposure, risk, and protective efficacy.

Vaccines aimed against malaria sporozoites might lead to a delayed acquisition of natural immunity, resulting in increased risk of malaria when the protection afforded by vaccination has waned compared with individuals who did not receive the vaccine. This was observed in other malaria interventions such as weekly malaria chemoprophylaxis and use of insecticide-treated bed nets. The phenomenon has been termed rebound malaria ^84, 85^. An additional three years of follow-up showed that RTS,S/AS01 vaccination might lead to periods of increased risk to uncomplicated malaria when vaccine-induced protection has waned ^85^. This observation is in line with the previous phase 2 study carried out in Kenya, where the initial protection provided by three RTS,S/AS01 doses was offset by rebound after five years in areas with higher-than-average exposure to malaria parasites ^86^. Three-year extension of the phase 3 trial in Nanoro, Burkina Faso showed an increase in clinical malaria incidence in the RTS,S vaccinees when the children were older. Nevertheless, the rebound was not outweighed by the initial benefit and there was no increased risk for severe malaria or a shift toward cerebral malaria ^85^.

## Novel adjunctive tools

Another important question that research would want to address is how to achieve and maintain stronger and longer-lasting VE through the use of adjuvants or optimal dose/delivery methods or both. An appropriate adjuvant will greatly increase the immunogenicity of a vaccine ^58, 87– 90^. Vaccine adjuvants have been tapped to target innate immune responses, activate the Toll-like receptor signaling pathway, and expand the antibody repertoire against the malaria parasite. Studies for novel and more effective human-compatible adjuvant to improve vaccine response are ongoing.

## Summary and Conclusions

Efforts to come up with efficacious malaria vaccine continue despite challenges. As new approaches (use of structural epitopes for antigen selection; development of novel vectors, adjuvants, and delivery methods; altered vaccine schedules and dosages; utilization of new tools) lend themselves to malaria research, a cooperative global effort should also be established to allow various vaccine candidates in the developmental pipeline to be compared side by side in proof-of-concept clinical trials to overcome most of the financial challenge in investing in large-scale good manufacturing practice (GMP) production, phase III and IV studies. It has become generally accepted that likely combinations of effective vaccines that complement each other will be used, and a successful vaccine must be able to induce both humoral and T-cell responses. But the efficacy of each vaccine component needs to be evaluated, or a hit-and-miss approach will entail extensive investment. The burden of developing a malaria vaccine is currently spread to only a few partners reluctant to invest as more candidates are discovered and innovations are made in delivery, yet there are knowledge gaps in the candidates’ mechanism/correlates of protection and undefined regulatory pathway(s). A collaborative effort is especially important to be initiated from the scientific community given that primary target groups are those in low- and middle-income countries. Finally, as illustrated above, most available data come from studies of *P. falciparum*, and studies on *P. vivax* are also needed to help advance vaccines that will contribute to malaria elimination.

## Acknowledgments

Authors thank their many collaborators—Sophie Houard and European Vaccine Initiative, Sodiomon B. Sirima and his team (Groupe de Recherche Action en Santé), Nobelpharma Co. Ltd.—for their ideas, motivation, and tenacity. An efficacious malaria vaccine can be achieved!
